# Intestinal Parasitic Infections in Marginalized Populations: Prevalence and Risk Factors Among the Bantar Community of Sunsari, Nepal

**DOI:** 10.1002/puh2.70275

**Published:** 2026-05-20

**Authors:** Mukesh Kumar Mahato, Jitendra Gautam, Niten Bharati, Darwin Niroula, Krishna Prasad Acharya, Tulsi Ram Gompo, Kishor Pandey

**Affiliations:** ^1^ Central Department of Zoology Institute of Science and Technology Tribhuvan University, Kirtipur Kathmandu Nepal; ^2^ Department of Livestock Services Animal Disease Investigation and Control Division Lalitpur Nepal; ^3^ Central Veterinary Laboratory Kathmandu Nepal

**Keywords:** awareness, intestinal parasites, prevalence, risk factors

## Abstract

**Background:**

Nepal continues to exhibit a high burden of intestinal parasite infections (IPIs). This study aimed to determine the prevalence and associated risk factors of IPIs among the indigenous Bantar community in Koshi Rural Municipality, Sunsari, Nepal.

**Methods:**

A total of 180 individuals aged 10–70 years were selected through convenience sampling. Stool samples were preserved in 2.5% potassium dichromate solution. Information on demographic and behavioral characteristics was gathered using a structured questionnaire. Three diagnostics techniques (direct wet mount, saturated salt flotation, and formal ether sedimentation) were employed to identify IPIs. Logistic regression analyses were conducted to determine association between the predictors and IPIs.

**Results:**

The overall prevalence of IPIs was 53.3% (*n* = 96). Helminthic infections accounted for 33.3% (*n* = 61), whereas protozoan infections were observed in 19.4% (*n* = 35). The most prevalent parasite was *Ascaris lumbricoides* (16%, *n* = 29), followed by *Giardia lamblia* (12.2%, *n* = 22), *Hymenolepis nana* (8.3%, *n* = 15), *Entamoeba histolytica* (7.2%, *n* = 13), *Strongyloides stercoralis* (6.7%, *n* = 12), and *Trichuris trichiura* (2.8%, *n* = 5). Notably, *E. histolytica* infection was exclusively found in females (14.4%, *n* = 13) and absent in males. Multivariate logistic regression identified nail‐biting behavior as a significant predictor of intestinal parasitic infections (adjusted odds ratio [aOR]: 2.75; 95% confidence interval [CI]: 1.12–6.9).

**Conclusions:**

These findings highlight a substantial parasitic burden in the Bantar community and suggest that lifestyle and hygiene‐related behaviors significantly contributed to infection risk. Targeted health education programs focusing on sanitation, personal hygiene, and food safety are urgently needed to mitigate the high prevalence of IPIs in this vulnerable population.

## Graphical abstract

Intestinal parasite infections remain highly prevalent (53.3%) among the Bantar community in Sunsari, Nepal, with *Ascaris lumbricoides* being the most common parasite and nail‐biting habit identified as a significant risk factor. The findings highlight the urgent need for targeted water, sanitation, hygiene, and health education interventions.

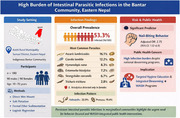

## 1 Background

An estimated 1.5 billion individuals suffer from intestinal parasite infections (IPIs), which are a persistent global public health concern, especially in tropical and subtropical areas with inadequate sanitation [1]. These infections, which are brought on by helminths (*Ascaris lumbricoides* and hookworms) and protozoa (*Giardia lamblia* and *Entamoeba histolytica*), are categorized by the World Health Organization (WHO) as neglected tropical diseases that prolong cycles of poverty, malnutrition, and stunted infant development [2, 3, 4]. Asia is higher illnesses caused by soil‐transmitted helminths due to dense populations, agricultural activities, and limited access to clean water [5, 6, 7]. Children and vulnerable people have the highest prevalence; in rural areas, rates might surpass 50% [8, 9]. This is made worse by open defecation, tainted water, and poor hygiene [10]. In low‐income nations, where reinfection rates are still dangerously high in the absence of integrated water, sanitation, and hygiene (WASH) measures, progress pauses despite widespread deworming programs [11, 12].

IPIs are endemic in Nepal, a lower middle‐income country with a varied geography ranging from Terai plains to Himalayan peaks, particularly in the Sunsari district and the southeast Terai region [13]. According to national studies, the prevalence is 15%–30% overall, whereas rates of 20%–50% are experienced by marginalized communities due to poverty, overcrowding, and monsoon‐flooded surroundings that promote fecal–oral transmission [14]. Risk factors that are exacerbated by climatic fluctuation and urbanization include barefoot walking, contact with untreated river water, and open defecation, which is performed by 40% of rural households, according to studies conducted in surrounding areas [15]. Due to social marginalization and landlessness, indigenous population is disproportionately affected [3, 16, 17].

Several studies investigated the prevalence of IPIs among ethnic groups, such as Magar, Sarki, Darai, and Bote, which was reported as 49.5%, 29.5%, 9.5%, and 29.5%, respectively [9, 13, 18].

A range of socioeconomic, demographic, behavioral, and environmental factors has been identified as contributors to IPIs’ transmission across different populations and regions. These include personal habits [5], level of knowledge [13], socioeconomic status [19], cultural, and bioenvironmental conditions [20]. In particular, poor hygiene practices, such as not using soap for handwashing before meals or after defecation [21], walking on barefoot [22], and nail‐biting habits [23], significantly increase the risk of IPIs. In children, WASH are directly linked to their nutritional status and prevalence of IPIs [24]. Additionally, cestode infection was primarily associated with unsafe slaughtering facilities [25].

The Bantar communities are an indigenous, marginalized people, predominantly living in southern Nepal's Terai area, with a population of approximately 61,500 [26]. They are traditionally farmers, laborers, and agricultural experts who frequently live in traditional bamboo and wood dwellings. They experience high rates of illiteracy and poverty [27]. By concentrating on this group, we fill a crucial evidence gap: Although generic Terai data are available, Bantar‐centric insights are lacking, which restricts customized public health interventions. This study aims to address several key research questions regarding IPIs in the Bantar community of Sunsari. First, it seeks to determine the species distribution and current prevalence of IPIs within this population, providing an updated epidemiological profile. Second, it investigates that environmental factors such as proximity to rivers and exposure to flooding and behavioral practices, including open defecation and consumption of raw or unwashed food, are the most significant predictors of infection. Finally, the study explores how socioeconomic conditions, cultural practices, and occupation‐related exposures specific to the Bantar community contribute to infection risk, with the goal of identifying insights that extend beyond existing research on other marginalized groups. Together, these questions aim to generate a more precise understanding of both the determinants and patterns of IPIs in this underserved population. During Nepal's efforts to achieve Sustainable Development Goal 6 on clean water and sanitation, this study not only estimates the burden but also identifies practical, ethnicity‐specific aspects that inform equitable measures.

## 2 Materials and Methods

### 2.1 Study Area

**FIGURE 1:**
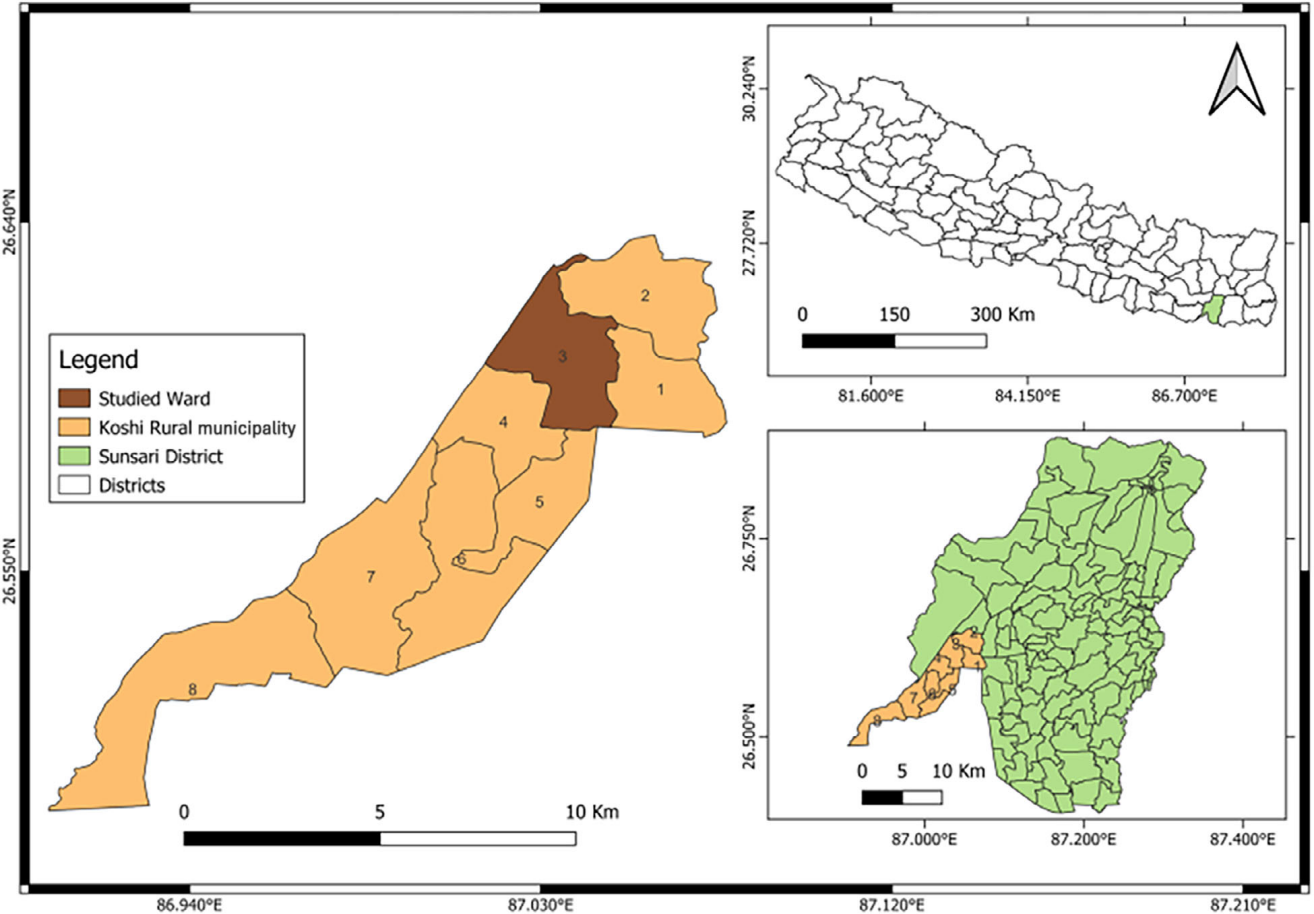
Map of Nepal showing study area.

The study was carried out in Koshi Rural Municipality, which is situated at coordinates 26.63° N and 87.06° E in the Sunsari District of Koshi Province, Nepal (Figure 1). About 522 km to the east of Nepal's capital, Kathmandu, is Sunsari District. About 48,804 people live in Koshi Rural Municipality, which is spread across eight administrative wards and occupies an area of 75.98 sq. km [28]. The municipality is in the Terai lowland region, which has a subtropical monsoon climate with hot, muggy summers (average temperature of 30–35°C) and mild winters (15–20°C). It also receives more than 2000 mm of rainfall annually. Numerous vector‐borne and parasitic diseases can thrive and spread under such ideal temperature and humidity levels. The region's population is diverse, comprising Bantar (an ethnic minority), Tharu, Rai, Musahar, and other groups of Terai descent. A sizable section of the local populace is made up of the Bantar community, which is frequently socioeconomically marginalized. With a literacy rate of roughly 72% and an average household size of 4.8 people, there are clear gender differences that favor men. Small‐scale trading, daily wage labor, livestock rearing, and subsistence farming are the main occupations.

### 2.2 Sampling

We sampled 90 male and 90 female participants using a convenience sampling technique from August to September 2021. A formal written consent was obtained before sampling, and participants under the age of 10 were excluded. Eligible participants were approached and informed about the study's objectives and procedures. Participants were instructed on proper fecal sample collection procedures, including the use of sterile wooden applicators during defecation. They were advised to avoid contamination of dirt or urine when collecting the sample into a sterile vial. Samples were collected the next morning, properly labeled, and stored in sterile ziplock containers. All fecal samples were preserved in a 2.5% (w/v) potassium dichromate solution and stored at 4°C. The samples were transported to the Central Department of Zoology, Tribhuvan University, Kathmandu, Nepal for further analysis.

### 2.3 Laboratory Examinations

#### 2.3.1 Wet Mount Technique

Approximately 2 g of each fecal sample was used for wet mount preparation for the Lugol's iodine test. A small portion of the sample was mixed with a few drops of iodine solution to enhance the visualization of cysts, eggs, and larvae. A small amount of this mixture was then placed on a clean glass slide, covered with a coverslip, and examined under a light microscope at high magnification (400×). The examination was carried out according to standard laboratory protocols described in previous investigations [29].

#### 2.3.2 Saturated Salt Flotation Technique

In a 15 mL centrifuge tube, 1 mL of the filtrate was mixed with 13 mL of sodium chloride (NaCl). The mixture was then centrifuged for 5 min at 1500 rpm. After centrifugation, the tubes were placed in test tube stands. Each tube was filled to the rim with the NaCl solution, forming a meniscus at the top. A coverslip was gently placed on the tube, ensuring it contacted the liquid surface. After allowing the preparation to stand undisturbed for 15–20 min, the coverslip was carefully removed and placed onto a glass slide for microscopic examination of diagnostic parasite stages [30].

#### 2.3.3 Formal Ether Sedimentation Technique

A total of 10 mL of 10% formal saline was added to 1 mL of filtrate in a 15 mL centrifuge tube. Subsequently, 3 mL of diethyl ether was added, and the mixture was shaken to ensure thorough mixing. The mixture was then centrifuged at 1500 rpm for 5 min. Following centrifugation, the supernatant was carefully discarded, and approximately two drops of sediment were mixed with Lugol's iodine on a glass slide. The slide was then examined under a microscope for the presence of parasitic stages [30].

#### 2.3.4 Questionnaire

A questionnaire was designed to effectively gather essential data to achieve the study's objectives, including sociodemographic information (age, gender, and family size) as well as lifestyle and behavioral characteristics of the Bantar community. These questionnaires addressed risk‐related behaviors, including walking barefeet, hand washing practices, nail trimming, sources of drinking water, and other relevant habits. The questionnaire was designed to effectively gather essential data to achieve the study's objectives.

#### 2.3.5 Data Analysis

Data were recorded, and preliminary cleaning was done in MS Excel before organizing the tables in MS Word. Statistical analyses were conducted using R (version 4.4.0) [31]. Descriptive statistics were performed on continuous variables such as ages and family members to calculate mean, standard deviations, and percentages, and basic frequency tables were constructed for categorical variables such as gender. Group differences were evaluated using *t*‐tests for continuous variables (e.g., age and family members) and chi‐square or Fisher's exact tests for categorical variables. Univariate logistic regression was used to estimate odds ratios (ORs) and 95% confidence intervals (CIs) for each potential predictor of infection. Any variables with estimated chi‐square *p* values < 0.2 in univariable analyses were included in a multivariate logistic regression model [31]. Model selection was performed using dredge function from the MuMIn package in R, which ranks models by Akaike information criteria (AIC). The best fitting model was adjusted odds ratios (aORs) and 95% CIs, providing insights into the independent effects of risk factors. aORs and their 95% CIs were calculated from the coefficients of the best fit logistic regression model.

## 3 Results

The mean age of male participants was significantly higher than that of female participants (32.3 vs. 26.7 years, respectively; *p* = 0.0008). In contrast, no significant difference was observed between the family sizes of both male and female participants (*p* = 0.099) (Table 1).

**TABLE 1:** Characteristic features of the study participants (*n* = 180).

Characteristics	Male (*n* = 90)	Female (*n* = 90)	*p* value	Total
**Demographic characteristics**	Mean (SD)	Mean (SD)		Mean (SD)
Age	32.3 (11.2)	26.7 (11.1)	0.0008***	29.49 (11.45)
No. of family members in home	4.64 (1.10)	4.38 (1.06)	0.099	4.5 (1.08)

*Note: p* values derived from independent *t*‐tests comparing males and females; *p < 0.05, **p < 0.01, ***p < 0.001; NS = not significant (*p* ≥ 0.05).

Abbreviation: SD, standard deviation.

Interestingly, compared to female participants, more men were illiterate who used to work in agriculture. However, other socioeconomic factors, such as household size, overcrowding, awareness of IPIs, and ownership of free‐ranging pigs and poultry, did not differ significantly by sex. Approximately two‐thirds of the participants were illiterate and lacked knowledge of IPIs. Despite these factors, most participants did not frequently report diarrhea or abdominal discomfort (Table 2).

**TABLE 2:** Socioeconomic characteristics of the study participants (*n* = 180).

Characteristics	Male (*n* = 90)	Female (*n* = 90)	*p* value	Total
**Socioeconomic characteristics**	*n* (%)	*n* (%)		*n* (%)
Can you read and write?				
Yes	22 (24.4)	48 (53.3)	0.0001*	70 (38.9)
No	68 (75.6)	42 (46.7)		110 (61.1)
Free‐ranging poultry or pig in house?				
Yes, we do have	17 (18.9)	21 (23.3)	0.513	38 (21.1)
No, we do not have	73 (81.1)	69 (76.7)		142 (78.9)
Do you have knowledge of IPIs’ prevention?				
Yes, we have some	9 (10)	16 (17.8)	0.558	25 (13.9)
No, we do not know about it	81 (90)	74 (82.2)		155 (86.1)
Do you feel frequent diarrhea or abdominal discomfort?				
Yes, I do feel so frequently	14 (15.6)	27 (30)	0.00003*	41 (22.8)
No, I do not feel it	76 (84.4)	63 (70)		139 (77.2)

*Note: p* values derived from chi‐square tests or Fisher's exact test for small, expected frequencies; *p < 0.05, **p < 0.01, ***p < 0.001; NS = not significant (*p* ≥ 0.05).

Abbreviation: IPI, intestinal parasitic infection.

**FIGURE 2:**
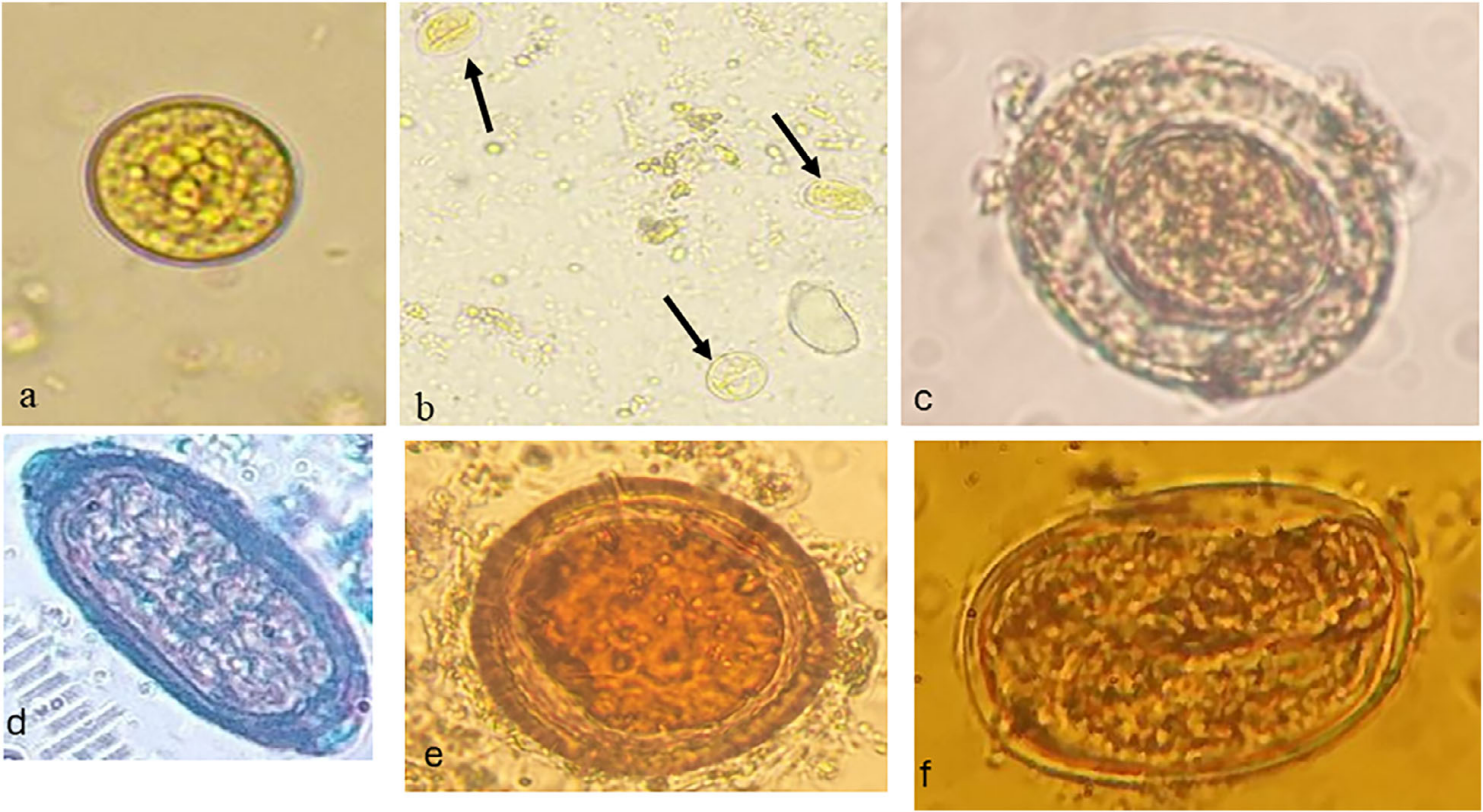
Microscopic images of different intestinal parasites detected on human stool samples. Protozoans ((a) *Entamoeba histolytica* and (b) *Giardia lamblia*) and helminths ((c) *Hymenolepis nana*, (d) *Trichuris trichiura*, (e) *Ascaris lumbricoides*, and (f) *Strongyloides stercoralis*).

Of the total 180 samples processed to visualize the eggs and larvae under microscope, more than half of the samples (53.3%, *n* = 96) contained eggs of one or more species of IPIs. Among the six‐parasite species eggs identified, two were protozoa (*E. histolytica* and *G. lamblia*), whereas the remaining four were helminths (*A. lumbricoides*, *Hymenolepis nana*, *Strongyloides stercoralis, and Trichuris trichiura*) (Figure 2).

*A. lumbricoides* had the highest prevalence of 16.1% (*n* = 29), followed by *G. lamblia* at 12.2% (*n* = 22). Other parasites identified included *H. nana* (8.3%, *n* = 15), *E. histolytica* (7.2%, *n* = 13), *S. stercoralis* (6.7%, *n* = 12), and *T. trichiura* (2.8%, *n* = 5). The overall prevalence of helminth infections was 33.9% (*n* = 61), which was higher than protozoan infections 19.4% (*n* = 35) (Table 3). Female participants showed a higher prevalence of *E. histolytica* compared to males; however, no other significant differences in parasite prevalence were observed between the sexes.

**TABLE 3:** Prevalence of intestinal parasites in participants (*n* = 180).

Parasite species	Male *n* = 90 (%)	Female *n* = 90 (%)	Chi‐square	*p* value	Total
**Helminth species**					
*Ascaris lumbricoides*	17 (18.9)	12 (13.3)	0.658	NS	29 (16.1)
*Hymenolepis nana*	11 (12.2)	4 (4.4)	2.618	NS	15 (8.3)
*Strongyloides stercoralis*	4 (4.4)	8 (8.9)	0.804	NS	12 (6.7)
*Trichuris trichiura*	5 (5.6)	0 (0)	NS	NS	5 (2.8)
**Protozoan species**					
*Entamoeba histolytica*	0 (0)	13 (14.4)	NS	**<0.001**	13 (7.2)
*Giardia lamblia*	5 (5.6)	17 (18.9)	6.266	**0.012**	22 (12.2)
Any infection	42 (46.7)	54 (60)	2.701	**NS**	96 (53.3)
Any helminth	37 (41.1)	24 (26.7)	3.571	**NS**	61 (33.9)
Any protozoan	5 (5.6)	30 (33.3)	20.43	**<0.001**	35 (19.4)

*Note: p* values derived from chi‐square tests or Fisher's exact test for small, expected frequencies; NS = not significant (*p* ≥ 0.05); *p < 0.05, **p < 0.01, ***p < 0.001. Bold indicates statistically significanct difference.

Abbreviation: NS, not significant.

There were no significant differences between men and women in terms of most of the behaviors reported (Table 4). However, higher proportion of men than women reported not using soap when washing their hands. Overall, most participants indicated poor hygiene practices, including not using soap for handwashing, not trimming their nails regularly, frequent nail‐biting, and not deworming in the past 6 months. Additionally, majority reported not covering cooked food and eating raw meat.

**TABLE 4:** Behavioral and lifestyle characteristics of the study participants (*n* = 180).

Characteristics	Male (*n* = 90)	Female (*n* = 90)	*p* value	Total
Do you use soap while washing your hands?				
Yes	7 (7.78)	24 (26.7)	0.001*	31 (17.2)
No	83 (92.2)	66 (73.3)		149 (82.8)
Did you trim your nails regularly every week?				
Yes	17 (18.9)	21 (23.3)	0.583	38 (21.1)
No	73 (81.1)	69 (76.7)		142 (78.9)
Do you wash raw eating vegetables and fruits?				
Yes	61 (67.8)	48 (53.3)	0.067	109 (60.6)
No	29 (32.2)	42 (46.7)		71 (39.4)
Do you wear foot ware while outdoors?				
Yes	75 (83.3)	71 (78.9)	0.567	146 (81.1)
No	15 (16.7)	19 (21.1)		34 (18.9)
Do you cover food after cooking/eating?				
Yes	76 (84.4)	80 (88.9)		156 (86.7)
No	14 (15.6)	10 (11.1)		24 (13.3)
Do you bite your Nails?				
Yes	59 (65.6)	56 (62.2)	0.756	115 (63.9)
No	31 (34.4)	34 (37.8)		65 (36.1)
Did you deworm within 6 months?				
Yes	18 (20)	20 (22.2)	0.855	38 (21.1)
No	72 (80)	70 (77.8)		142 (78.9)
Do you consume raw/uncooked meat?				
Yes	5 (5.56)	4 (4.44)	0.0001*	9 (5)
No	85 (94.4)	86 (95.6)		171 (95)

*Note: p* values derived from chi‐square tests or Fisher's exact test for small, expected frequencies (e.g., raw/uncooked meat); *p < 0.05, **p < 0.01, ***p < 0.001; NS = not significant (*p* ≥ 0.05).

Logistic regression analysis showed that eating raw vegetables without washing (OR: 3.4, 95% CI: 1.8–6.5), covering food after cooking and eating (OR: 1.4, 95% CI: 0.7–2.9), and biting nails (OR: 4.5, 95% CI: 2.3–8.8) were significantly associated with a higher prevalence of IPIs in univariate analysis. Multivariate analysis further identified biting nails as a strong independent risk factor (Table 5) (aOR: 2.8, 95% CI: 1.1–6.9). Other behavioral factors, such as covering food after cooking and eating, were not significantly associated with infection.

**TABLE 5:** Prevalence and odds ratio of IPIs with respect to behavioral characteristics using logistic regression analysis (*n* = 180).

Characteristics	%	Univariate OR	Multivariate aOR
		(95% CI)	(95% CI)
Gender			
Male	42	0.7 (0.4–1.2)	2.0 (0.8–5.3)
Female	56	Ref	Ref
Household crowding			
Live ≤ 4 members in house	54	Ref	
Live > 4 members in house	46	0.7 (0.4–1.3)	
Can you read and write?			
Yes	44	0.7 (0.4–1.2)	
No	55	Ref	
Free‐ranging poultry or pig in house			
No, we do not have	49	Ref	
Yes, we do have	55	1.3 (0.6–2.6)	
Do you have knowledge of IPIs’ prevention?			
Yes, we have some	56	1.3 (0.6–3.0)	
No, we do not know about it	50	Ref	
**Behavioral characteristics**			
Do you use soap while washing your hands?			
No	52	1.3 (0.6–2.8)	
Yes	45	Ref	
Did you trim your nails regularly every week?			
No	50	Ref	
Yes	60	0.7 (0.2–2.5)	
Do you wash raw eating vegetables and fruits?			
No	62	**3.5 (1.8–6.5)**	
Yes	32	**Ref**	
Do you walk barefeet while outdoors?			
No	52	1.4 (0.7–2.9)	
Yes	44	Ref	
Do you cover food after cooking/eating?			
No	29	**Ref**	Ref
Yes	54	**1.4 (0.7–2.9)**	2.3 (0.9–6.8)
Do you bite your nails?			
No	28	**Ref**	**Ref**
Yes	64	**4.5 (2.3–8.8)**	**2.75 (1.1–6.9)**
Did you deworm within 6 months?			
Yes	47	0.9 (0.4–1.7)	
No	51	Ref	
Do you consume raw/uncooked meat?			
No	47	Ref	Ref
Yes	67	2.2 (1.0–5.2)	0.9 (0.3–2.4)
Do you play with soils?			
No	47	Ref	
Yes	55	1.4 (0.8–2.5)	
Do you drink boiled water?			
No	52	Ref	
Yes	47	0.8 (0.4–1.6)	

*Note:* Univariate and multivariate logistic regression analyses were performed; multivariate model was adjusted for significant univariate factors. Significant associations (*p* < 0.05) are bolded, *p < 0.05, **p < 0.01, ***p < 0.001.

Abbreviations: aOR, adjusted odds ratio; CI, confidence interval; OR, odds ratio.

## 4 Discussion

This study highlights the widespread presence of IPIs within the Bantar community in eastern Nepal, with a recorded prevalence of 53.3%. However, significantly higher rates have been reported in other communities, which might be due to differences in study design and location where they were conducted. For example, Yadav [32] documented 81% prevalence among the Musahar community in Balan Bihuli, Saptari, Nepal. Similarly, an extremely high prevalence of 97% was reported among the indigenous Chepang community in central Nepal [33]. In contrast, lower prevalence rates have been reported in other regions and communities: 27.3% among the Meche community at Jhapa [34], and 28.63% was reported among the indigenous Satar and Chaudhary communities in Birtamode Municipality, Jhapa, Nepal, whereas 23.3% was reported among Tibeto‐Burman, Indo‐Aryan, and Dalit schoolchildren in the Chitwan district, 24.1% among slum population at Kaski [35], 28.6% in the Satar and Chaudhary communities of Jhapa [5]; 17% in the Kumal community in Chitwan [16], 36.6% among Chepang and Musahar communities of Makwanpur and Nawalparasi [36], 41.1% in the Squatter community in Sunsari [37], and 28% among the Badi community in Surkhet [3]. Additionally, the prevalence was 31.32% among the Sarki ethnic group in Pala Rural Municipality, Baglung, and 33.3% among the Musahar community in the Makwanpur and Nawalparasi districts. The variation in IPIs’ prevalence across these studies may be attributed to differences in climatic conditions, sanitation infrastructure, and levels of awareness regarding personal hygiene and parasitic transmission.

Environmental, social, infrastructural, and behavioral factors interact in a complex way to cause variations in the prevalence of IPIs between studies in Nepal, especially among vulnerable people [1, 8]. Due to ingrained vulnerabilities, underprivileged groups like Dalits, indigenous Tharu, Badi, and squatter populations consistently exhibit 20%–50% [1, 3] prevalence higher than the usual 15%–30% even though national deworming operations have lowered overall rates since 2003 [38].

Studies in Nepal's marginalized populations have found that the prevalence of IPIs varies from 20% to 50% [38, 39]. These variations are mostly caused by variations in the country's climate, sanitation facilities, and levels of hygiene awareness [7]. In contrast to colder hills (15%–25%), Terai locations like Sunsari have higher rates (30%–40%) because of monsoon flooding and humidity, which encourage helminth survival [40]. Elevated transmission is caused by sanitation gaps, such as 40% open defecation in rural and slum regions compared to those with better access to toilets [24, 41]. Low hygiene awareness, which shows up as inadequate handwashing, barefoot walking, and low deworming uptake, increases risks two to seven times, especially for ethnic minorities who encounter cultural barriers [35, 42]. Exposure to contaminated water and soil is further increased by socioeconomic variables, including poverty, riverfront settlements, and jobs like farming or scavenging [7, 10, 21].

In this study, female (60%) had higher prevalence of IPI compared to males (46.7%), and the finding was found to be similar with following studies, which also stated that females had a higher prevalence [18, 21, 26]. It may be due to their frequent participation in domestic tasks like food preparation, cleaning, and childcare, which may expose them to more contaminated water or soil; females may have a higher infection rate. Furthermore, this discrepancy may also be caused by behavioral and sociocultural factors, such as women in rural communities being less likely to seek medical attention and being more vulnerable to nutritional issues. However, some of the previous studies showed that higher in male such as [11, 42, 43, 44, 45]. These discrepancies could result from variations in the study population, sample size, lifestyle, or geographic distribution. Males may be more likely to work outdoors in some environments, such as farming and cattle handling, which increases their exposure to parasite eggs and larvae in contaminated areas. Therefore, it seems that context‐dependent behavioral and environmental factors influence gender‐related differences in infection rates.

It was discovered that there were more helminthic parasites (33.3%, *n* = 61) than protozoan parasites (19.4%, *n* = 35), which was comparable to earlier research like [13, 46, 47, 48]. Poor sanitation, open defecation, and insufficient deworming coverage may be the cause of the persistence of helminthic infections, especially in low‐income and rural areas. On the other hand, a greater percentage of protozoan infections have been reported in some Nepali studies, including [11, 40, 45, 49, 50]. The use of tainted drinking water and the fecal‐oral transmission pathway of protozoa, such as *E. histolytica* and *G. lamblia*, may be the cause of this discrepancy. Furthermore, the prevalence of one parasite group over another may also be influenced by regional sanitation practices, seasonal variation, and diagnostics’ techniques.

*A. lumbricoides* was the most common parasite in this study (16%), which was similar with following those studies [11, 51, 52]. Around the world, *A*. *lumbricoides* infections are extremely prevalent. It rose in areas with inadequate sanitation, especially those where children urinate on the ground and human waste is used as fertilizer [3, 7, 53, 54]. *A. lumbricoides* was found to be the most prevalent intestinal parasite (16%) in this investigation, which is in line with the results of other studies [11, 51, 52]. The high frequency of *A. lumbricoides* suggests that soil‐transmitted helminths continue to pose a serious threat to Nepal's public health. One of the most common parasitic diseases in the world is *A. lumbricoides* infection, especially in underdeveloped areas with inadequate sanitation and hygiene. Ingestion of infectious eggs from tainted food, water, or soil is frequently associated with the transmission. In places where children regularly play on contaminated ground, where open defecation is common, and where untreated human feces are used as fertilizer, its prevalence tends to increase [23, 43]. All these elements work together to make *A. lumbricoides* infections persistent, particularly in who reside in rural and low‐resource areas.

The overall prevalence of specific parasites was generally similar between males and females, with notable exception of *E. histolytica*, which was more prevalent among females. This trend aligns with previous studies that reported higher prevalence in females, potentially due to cultural practices that assign women soil‐related household chores and agricultural work [29, 34, 55]. However, other studies, such as those conducted in Darai community [56] and among the Kumal indigenous group in Nepal [16], found no significant difference in risk between sexes. In contrast, research among indigenous communities in rural Peninsular Malaysia [57] and rural Guinea, Africa [58] reported lower IPIs prevalence in females.

Additional studies are necessary to further investigate and confirm these relationships. Among the various symptoms and behavioral or lifestyle factors analyzed for their associations with IPIs, biting nails showed the strongest correlation, exhibiting the highest OR in both the univariate and multivariate models (aOR: 2.57 [1.121–6.9274]). Similar patterns have been observed in earlier studies such as [11, 13, 59]. Notably, eating raw vegetables before eating and covering of food after cooking also have been identified as a significant risk factor for IPIs in multiple studies, including those conducted among children from squatter communities in Dharan Municipality [47]. Comparable findings have also been reported in other ethnic groups such as the Tharu, Satar, and Chaudhary communities, as well as the Chepang community in Chitwan [40, 41, 43]. Despite long‐standing government initiatives, Nepal's attempts to eradicate soil‐transmitted helminths and other enteric parasites continue to fall short, as evidenced by the study's finding that the Bantar community had a high prevalence of IPIs. Initiated in 2004 and expanded through school‐based mass drug administration, Nepal's National Deworming Program (NDP) seeks to provide periodic albendazole/mebendazole to children ages 1–19 in endemic districts [39]. High coverage of the program has been observed in a variety of contexts, including formal education systems. However, it is unlikely that school‐centric deworming platforms will fairly benefit underprivileged communities like the Bantar community, who often have low school attendance and high socioeconomic exclusion rates [60]. This could help to explain why our group had higher than average infection prevalence [3].

In addition, Nepal's WASH sector strategic plans, like the WASH in Schools initiative and the Sanitation and Hygiene Master Plan (2011–2030) [52], place a strong emphasis on promoting hygiene, better sanitation, and universal access to clean water. These regulations have not always been translated into community‐level changes, though, with rural and marginalized settlements disproportionately missing access to clean drinking water, sanitary facilities, and persistent behavior modification initiatives [24]. Our discovery that contaminated water sources and insufficient access to latrines were substantially linked to increased infection rates emphasizes the necessity of more firmly integrating WASH investments into the management of parasitic diseases. Interestingly, the present NDP does not routinely combine deworming efforts with family or community WASH programs [5].

To best of our knowledge, the study provides the first comprehensive analysis of IPIs among the Bantar indigenous community, though there are some point prevalence studies already conducted before. Yet, the study being cross‐sectional has some limitations that fail to capture temporality, such as seasonal changes that could contribute to prevalence of IPI. The small, convenience‐based sampling might reduce statistical power and generalizability.

Although there are several limitations to be aware of, this study offers important findings regarding IPIs in a community that has not received enough attention. The causal interpretation of observed relationships is limited by the cross‐sectional design. Microscopy and a single stool sample might have misclassified illnesses and understated frequency. Recall and social desirability bias might affect self‐reported behavioral data. Important variables were not evaluated, including immunosuppression, seasonal fluctuation, dietary status, and previous health education. It is possible that conclusions drawn from the Bantar community in Sunsari cannot be applied to other Nepali populations. Lastly, conclusions on the efficacy of the strategy were limited because the study did not explicitly assess the coverage or caliber of deworming and WASH program implementation.

## 5 Conclusions

Despite ongoing deworming efforts, 53.3% of the individuals in the Bantar community had IPIs. Limited use of anthelmintic medication in the past 6 months and poor awareness of IPIs indicate that current interventions have had limited impact on improving public knowledge. Risky hygiene behaviors, including nail‐biting, irregular nail trimming, not washing raw fruits and vegetables, and failing to cover food, were common, with nail biting identified as key risk factor for multivariate analysis. These findings highlight the need for targeted health education emphasizing hygiene and lifestyle improvement. Further research across the Bantar and other Nepali communities is essential to strengthen evidence, inform policy, and enhance public health initiatives.

## Author Contributions

Conceptualization: Mukesh Kumar Mahato and Kishor Pandey. Methodology: Mukesh Kumar Mahato, Jitendra Gautam, Niten Bharati, and Kishor Pandey. Software: Niten Bharati and Tulsi Ram Gompoo. Validation: Kishor Pandey. Writing – original draft preparation: Mukesh Kumar Mahato, Jitendra Gautam, Niten Bharati, and Kishor Pandey. Writing – review and editing: Mukesh Kumar Mahato, Jitendra Gautam, Niten Bharati, Darwin Niroula, Krishna Prasad Acharya, Tulsi Ram Gompoo, and Kishor Pandey. Supervision: Kishor Pandey. All the authors have read and agreed to the published version of the manuscript.

## Funding

This research received provincial funding from the Nepal Health Research Council.

## Ethics Statement

The Nepal Health Research Council granted ethical permission for this study (approved no. 586/2021 P).

## Consent

Informed consent was obtained from the participants before the sample collection.

## Conflicts of Interest

The authors declare no conflicts of interest.

## Data Availability

Data supporting the findings of this study are available from the corresponding author upon reasonable request.
